# Estimated plasma volume status and the risk of 30-day mortality among patients with traumatic brain injury: analysis from the MIMIC-IV database

**DOI:** 10.3389/fneur.2026.1893304

**Published:** 2026-09-14

**Authors:** Qinpu Qin, Wenting Kang, Qing Ban, Ye Li, Xin Guan

**Affiliations:** Department of Neurosurgery, Xuanwu Hospital Capital Medical University, Beijing, China

**Keywords:** 30-day mortality, estimated plasma volume status, KH-ePVS, MIMIC-IV, traumatic brain injury

## Abstract

**Background:**

Plasma volume status (PVS) is an indicator of fluid status, and fluid balance is linked to the prognosis of critically ill patients. This analysis aimed to examine the association of estimated PVS (ePVS) with mortality risk among traumatic brain injury (TBI) patients.

**Methods:**

This retrospective cohort analysis extracted TBI patient data from the Medical Information Mart for Intensive Care-IV (MIMIC-IV) database spanning 2008–2022. ePVS was calculated using the Duarte formula (Duarte-ePVS) and the Kaplan–Hakim formula (KH-ePVS), respectively. The associations of Duarte-ePVS and KH-ePVS with 30-day mortality risk among TBI patients were examined *via* Cox regression analyses. The non-linear associations of ePVS with 30-day mortality were analyzed *via* the restricted cubic splines (RCS) curve.

**Results:**

The analysis cohort comprised 1,440 TBI patients, survival outcomes showed 247 (17.15%) deaths and 1,193 (82.85%) survivors at 30-day follow-up. A 1-SD (standard deviation) increase in KH-ePVS levels [HR (95%CI) = 1.17 (1.02–1.34)] and KH-ePVS levels ≥11.458 [HR = 1.43 (1.06–1.95)] were linked to an elevated risk of 30-day mortality. No significant relationships of high Duarte-ePVS levels and Duarte-ePVS levels ≥7.134 with 30-day mortality risk were found (*P* > 0.05). No significant nonlinear association between KH-ePVS levels and 30-day mortality risk was found (*P* > 0.05).

**Conclusion:**

Elevated KH-ePVS levels were associated with an increased risk of short-term mortality among TBI patients, indicating a potential role for KH-ePVS levels in risk assessment.

## Introduction

Traumatic brain injury (TBI) refers to the structural or physiological damage to brain function caused by external forces, typically resulting from falls, motor vehicle collisions, or assaults (1). TBI is a major cause of death and disability, imposing a heavy disease burden worldwide (2, 3). TBI occurs in 50–60 million people each year, and each TBI death is associated with approximately 24 years of life lost (2, 4). TBI patients transferred to the intensive care unit (ICU) have a high incidence of complications and short-term mortality (5–7). The main risk factors for poor prognosis in TBI patients involve age, severity of initial injury, increased intracranial pressure, and secondary injuries (cerebral edema, impaired perfusion, and hypoxia) (1, 2). Although advances in disease care, identifying factors that influence prognosis may aid in disease risk stratification and individualized treatment.

Fluid balance is critical in the management of ICU patients. Fluid overload can lead to poorer survival outcomes in critically ill patients, while implementing interventions to restrict positive cumulative fluid balance can improve patient prognosis (8, 9). Plasma volume (PV) constitutes the liquid component of circulating blood and is critical for maintaining vascular tension, tissue perfusion, and inflammatory homeostasis (10). PV can reflect fluid balance status, and an increase in PV is usually correlated with fluid overload (11). Several studies have reported that high PV load may exacerbate brain damage among TBI patients through excessive activation of the renin-angiotensin-aldosterone system (12–14). PV is usually measured using tracer dilution techniques, but these methods are expensive, time-consuming, and complex (10). Estimated plasma volume status (ePVS) based on hemoglobin and hematocrit (Duarte-ePVS) (15), and Kaplan–Hakim ePVS (KH-ePVS) based on hematocrit, body weight, and gender were proposed (16). These ePVS values were found to have similar effects to PV measured by tracer dilution techniques (15, 16). A recent study reported that high Duarte-ePVS and KH-ePVS levels were related to an increased risk of short-term mortality among intracerebral hemorrhage patients (17). However, the influence of ePVS levels on the prognosis of TBI patients has not been reported. Thus, this analysis aimed to explore the association between ePVS levels and mortality risk among TBI patients based on large sample data from a public database to assist in the clinical management and risk stratification of TBI patients.

## Methods

### Study design and patients

Data utilized in this retrospective cohort analysis were sourced from the Medical Information Mart for Intensive Care-IV (MIMIC-IV) database covering 2008–2022. MIMIC-IV records real inpatient data from patients admitted to a tertiary academic medical center (Boston, MA, USA), with data collection beginning in 2008 and currently updated to 2022 (https://mimic.mit.edu/docs/iv/about/). MIMIC-IV database collects demographic information, laboratory measurements, medications, vital signs, and mortality information for ICU patients. Ethical approval for the MIMIC database was granted by the Institutional Review Boards of the Beth Israel Deaconess Medical Center and the Massachusetts Institute of Technology. No informed consent was required as all patient records were fully anonymized. The analysis cohort comprised TBI cases at first ICU admission. The following exclusion criteria were applied to patient selection: (1) age < 18 years old; (2) the length of ICU stay < 24 h; (3) those diagnosed with polycythemia; (4) those with missing hemoglobin data; (5) those with missing information on weight; and (6) those with missing data on survival status. TBI patients were identified in the MIMIC database using the International Classification of Diseases (ICD-9/10) codes (ICD-9: 850- to 854-; ICD-10: S06, S060- to S066-).

### Outcomes

The outcome of this analysis was 30-day mortality, which refers to the death of a patient from any cause within 30 days after ICU admission. In this analysis, patient survival status was recorded up to 30 days after ICU admission, with in-hospital survival data obtained from hospital records and out-of-hospital survival data extracted from Social Security Administration records.

### Calculation of ePVS

The exposure variable in this analysis was ePVS. The calculation of ePVS was carried out using the Strauss-derived Duarte formula (Duarte-ePVS) (15) and the Kaplan–Hakim formula (KH-ePVS) (16), respectively. Duarte-ePVS was calculated as:

Duarte-ePVS = 100 × [1 – hematocrit] / hemoglobin (g/dL) where hematocrit is a fraction. KH-ePVS was calculated as: KH-ePVS = [(aPV – iPV) / iPV] × 100% where aPV = [1 - hematocrit] × [a + (b × weight (kg))] and the parameters of a and b are: (males: a = 1,530, b = 41; females, a = 864, b = 47.9); iPV = c × weight (kg) and the parameter of c is: (males: c = 39; females: c = 40). In the analysis, the classification of Duarte-ePVS (< 7.134, ≥7.134) and KH-ePVS (< 11.458, ≥11.458) was determined according to the optimal cutoff value using the “surv_cutpoint” method in the “survminer” package.

### Data collection

Initial ICU admission records of patients were collected, containing age, gender, race, fluid balance within 24 h, Sequential Organ Failure Assessment (SOFA), Glasgow Coma Scale (GCS) score, lesion type, heart failure, acute myocardial infarction (AMI), sepsis, acute kidney injury (AKI), hematological system diseases, weight, heart rate, oxyhemoglobin saturation (SpO_2_), systolic, diastolic, respiratory rate, temperature, white blood cell (WBC), platelet, hemoglobin, red blood cell distribution width (RDW), hematocrit, creatinine, international normalized ratio (INR), blood urea nitrogen (BUN), glucose, calcium, anion gap, ventilation, vasopressor, neurosurgical intervention, mannitol, diuretic, length of ICU stay, and 30-day follow-up time. The 24-h fluid balance was calculated as 24-h fluid intake minus 24-h fluid output. AMI, heart failure, and hematological system diseases were determined *via* ICD-9/10 codes. Sepsis diagnosis followed Sepsis 3.0 criteria, requiring both suspected infection and a SOFA score ≥2 (18). AKI diagnosis followed KDIGO criteria (19). Polycythemia diagnostic criteria required hemoglobin >16.0 g/dL (females) or >16.5 g/dL (males), alternatively hematocrit exceeding 48% (females) or 49% (males) (20). Neurosurgical intervention included craniotomy and minimally invasive surgery.

### Statistical analysis

Continuous variables' normality was assessed using skewness and kurtosis measures, with variance homogeneity checked by Levene's test. Continuous variables were shown as mean ± SD (standard deviation) or median and quartiles [M (Q1, Q3)], with group comparisons performed using *t*-test, *t*'-test, or Wilcoxon rank-sum test where applicable. Categorical variables were presented as counts and percentages [*n* (%)], with group comparisons conducted utilizing the Chi-square test or the Fisher exact test. Covariates with ≥20% missing data were removed, whereas those with < 20% underwent missing value imputation using random forest interpolation. Comparative assessments of variable differences before vs. after data imputation were conducted (Supplementary Table 1, Supplementary Figure 1).

Confounders related to 30-day mortality were first screened *via* univariable Cox analysis, then bidirectional stepwise regression applying Akaike Information Criterion (AIC) minimization criteria was utilized to screen for variables with *P* < 0.05 in univariable analysis (Supplementary Table 2). The associations of Duarte-ePVS and KH-ePVS with 30-day mortality among TBI patients were evaluated *via* univariable and multivariable Cox regression analyses, with associations presented as hazard ratio and 95% confidence interval [HR (95%CI)]. Age, race, SOFA, GCS, hematological system diseases, heart rate, temperature, WBC, RDW, BUN, glucose, ventilation, vasopressor, and mannitol were adjusted in the multivariable analyses. The non-linear associations of Duarte-ePVS and KH-ePVS with 30-day mortality were investigated *via* the restricted cubic splines (RCS) curve. The Kaplan-Meier (KM) curves were employed to evaluate the influence of Duarte-ePVS and KH-ePVS on 30-day survival. Furthermore, additional analyses of the ePVS and 30-day mortality association were conducted in age, gender, heart failure, sepsis, AKI, and hematological system diseases subgroups. R 4.4.3 software (Vienna, Austria) was employed for all analyses, with *P* < 0.05 considered significant.

## Results

### Patient characteristics

A total of 2,821 patients with TBI were identified from the MIMIC-IV database. After applying exclusion criteria, 1,381 patients were removed, leaving 1,440 eligible patients for final analysis (Figure 1). Comparison of characteristics between the included and excluded cohorts revealed significant differences across multiple variables (Supplementary Table 3). Among 1,440 enrolled TBI patients, survival outcomes showed 247 (17.15%) deaths and 1,193 (82.85%) survivors at 30-day follow-up. Table 1 lists the characteristics of TBI patients. Population characteristics showed 762 (52.92%) patients aged ≥65 years, 901 (62.57%) males, and 872 (60.56%) patients of White race. The mean values for Duarte-ePVS and KH-ePVS were 6.01 (±1.79) and 1.22 (±11.54), respectively. The median length of ICU stay was 2.86 (1.74, 5.95) days. The 30-day mortality group presented significant differences from 30-day survival group in terms of age, race, SOFA, GCS score, heart failure, sepsis, AKI, hematological system diseases, weight, heart rate, diastolic, temperature, WBC, platelet, hemoglobin, RDW, hematocrit, creatinine, INR, BUN, glucose, anion gap, ventilation, vasopressor, mannitol, diuretic, length of ICU stay, 30-day follow-up time, Duarte-ePVS, and KH-ePVS (*P* < 0.05).

**Figure 1:**
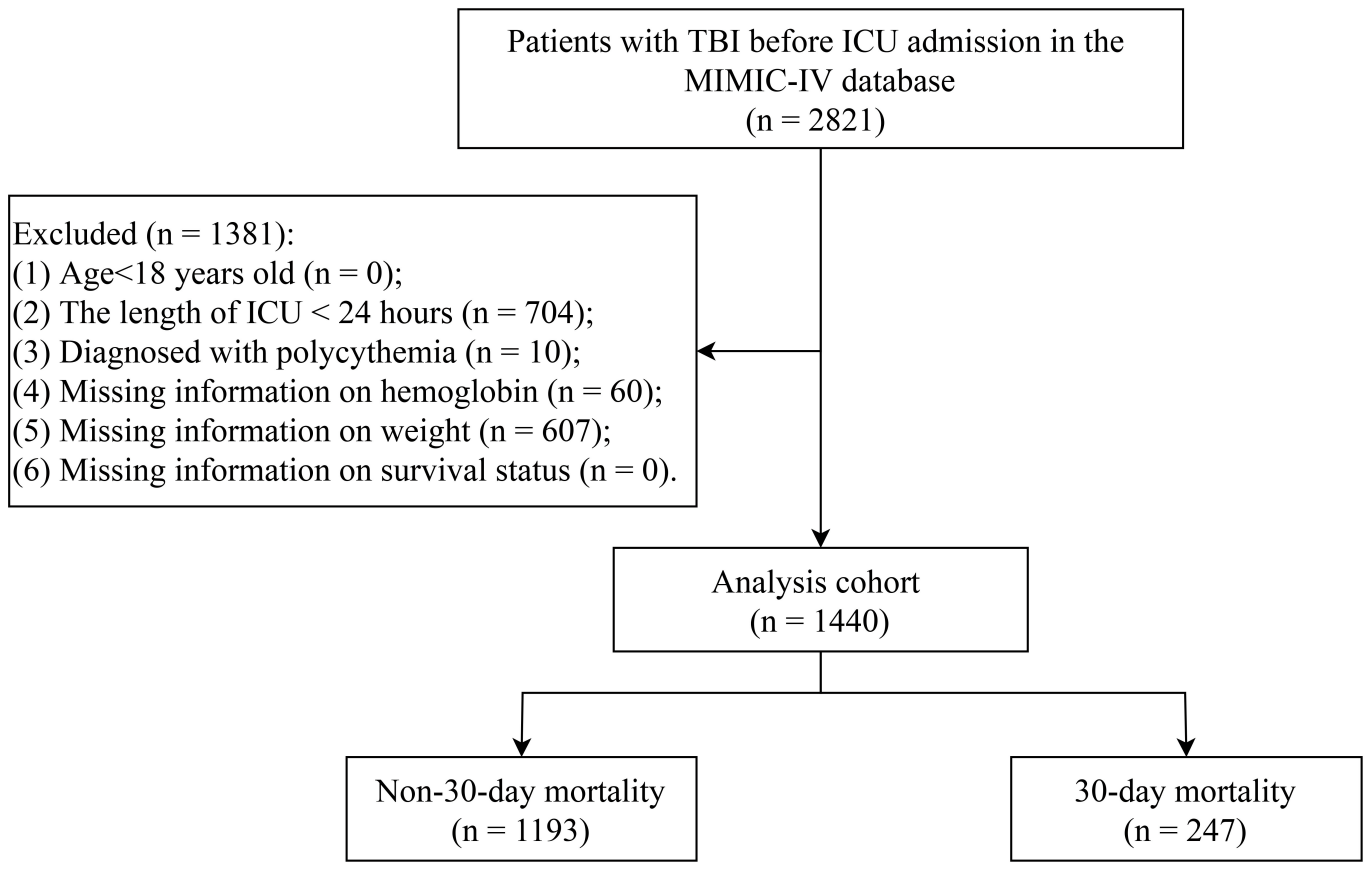
Screening flowchart for TBI patients. TBI, traumatic brain injury; ICU, intensive care unit; MIMIC-IV, the medical information mart for intensive care-IV.

**Table 1:** Characteristics of patients with traumatic brain injury (TBI).

Variables	Total (*N* = 1,440)	30-day follow-up		*P*
		Survivors (*N* = 1193)	Non-survivors (*N* = 247)	
Age, years, *n* (%)				< 0.001
< 65	678 (47.08)	602 (50.46)	76 (30.77)	
≥65	762 (52.92)	591 (49.54)	171 (69.23)	
Gender, *n* (%)				0.191
Male	901 (62.57)	756 (63.37)	145 (58.70)	
Female	539 (37.43)	437 (36.63)	102 (41.30)	
Race, *n* (%)				< 0.001
White	872 (60.56)	741 (62.11)	131 (53.04)	
Black	79 (5.49)	71 (5.95)	8 (3.24)	
Others	179 (12.43)	159 (13.33)	20 (8.10)	
Unknown	310 (21.53)	222 (18.61)	88 (35.63)	
Fluid balance within 24 h, *n* (%)				0.703
≤ 0	299 (20.76)	245 (20.54)	54 (21.86)	
>0	1,141 (79.24)	948 (79.46)	193 (78.14)	
SOFA, score, Mean (±SD)	3.56 (±2.58)	3.23 (±2.35)	5.14 (±3.04)	< 0.001
GCS, *n* (%)				0.003
≥13	1,106 (76.81)	935 (78.37)	171 (69.23)	
< 13	334 (23.19)	258 (21.63)	76 (30.77)	
Lesion type, *n* (%)				0.163
Traumatic subdural hemorrhage	464 (32.22)	388 (32.52)	76 (30.77)	
Traumatic subarachnoid hemorrhage	211 (14.65)	181 (15.17)	30 (12.15)	
Traumatic subarachnoid hemorrhage/traumatic subdural hemorrhage	169 (11.74)	139 (11.65)	30 (12.15)	
Focal brain injury	139 (9.65)	121 (10.14)	18 (7.29)	
Intracranial injury of other or unspecified nature	457 (31.74)	364 (30.51)	93 (37.65)	
Heart failure, *n* (%)				< 0.001
No	1,280 (88.89)	1,082 (90.70)	198 (80.16)	
Yes	160 (11.11)	111 (9.30)	49 (19.84)	
AMI, *n* (%)				0.193
No	1,402 (97.36)	1,165 (97.65)	237 (95.95)	
Yes	38 (2.64)	28 (2.35)	10 (4.05)	
Sepsis, *n* (%)				0.004
No	1,179 (81.88)	993 (83.24)	186 (75.30)	
Yes	261 (18.12)	200 (16.76)	61 (24.70)	
AKI, *n* (%)				< 0.001
No	807 (56.04)	697 (58.42)	110 (44.53)	
Yes	633 (43.96)	496 (41.58)	137 (55.47)	
Hematological system diseases, *n* (%)				0.008
No	846 (58.75)	720 (60.35)	126 (51.01)	
Yes	594 (41.25)	473 (39.65)	121 (48.99)	
Weight, kg, Mean (±SD)	76.91 (±19.69)	77.54 (±19.22)	73.85 (±21.60)	0.007
Heart rate, bpm, Mean (±SD)	85.41 (±18.75)	84.83 (±18.12)	88.20 (±21.37)	0.022
Systolic, mmHg, Mean (±SD)	131.34 (±22.98)	131.52 (±22.33)	130.43 (±25.94)	0.539
Diastolic, mmHg, Mean (±SD)	72.42 (±17.03)	72.92 (±16.72)	70.04 (±18.32)	0.016
Respiratory rate, bpm, Mean (±SD)	18.60 (±4.87)	18.53 (±4.74)	18.96 (±5.42)	0.245
SPO_2_, %, Mean (±SD)	97.65 (±3.02)	97.61 (±2.95)	97.86 (±3.31)	0.229
Temperature, °C, Mean (±SD)	36.79 (±0.84)	36.86 (±0.72)	36.48 (±1.24)	< 0.001
WBC, K/uL, M (Q1, Q3)	10.50 (7.80, 13.70)	10.30 (7.60, 13.50)	11.90 (8.95, 16.20)	< 0.001
Platelet, K/uL, M (Q1, Q3)	189.00 (145.00, 241.00)	190.00 (149.00, 242.00)	177.00 (130.50, 230.50)	0.025
Hemoglobin, g/dL, Mean (±SD)	11.47 (±2.01)	11.59 (±1.95)	10.89 (±2.17)	< 0.001
RDW, %, Mean (±SD)	14.15 (±1.82)	13.98 (±1.68)	14.94 (±2.21)	< 0.001
Hematocrit, %, Mean (±SD)	34.48 (±5.74)	34.75 (±5.56)	33.16 (±6.39)	< 0.001
Creatinine, mg/dL, M (Q1, Q3)	0.80 (0.70, 1.10)	0.80 (0.70, 1.00)	1.00 (0.70, 1.30)	< 0.001
INR, ratio, Mean (±SD)	1.23 (±0.24)	1.21 (±0.22)	1.32 (±0.31)	< 0.001
BUN, mg/dL, Mean (±SD)	18.31 (±11.87)	17.24 (±10.69)	23.49 (±15.43)	< 0.001
Glucose, mg/dL, Mean (±SD)	136.62 (±51.29)	132.63 (±47.78)	155.89 (±62.29)	< 0.001
Calcium, mg/dL, Mean (±SD)	8.42 (±0.76)	8.43 (±0.73)	8.34 (±0.88)	0.125
Anion gap, mEq/L, Mean (±SD)	14.53 (±3.60)	14.35 (±3.48)	15.39 (±4.03)	< 0.001
Ventilation, *n* (%)				< 0.001
No	304 (21.11)	281 (23.55)	23 (9.31)	
Yes	1,136 (78.89)	912 (76.45)	224 (90.69)	
Vasopressor, *n* (%)				< 0.001
No	1,186 (82.36)	1,030 (86.34)	156 (63.16)	
Yes	254 (17.64)	163 (13.66)	91 (36.84)	
Neurosurgical intervention, *n* (%)				0.102
No	1,338 (92.92)	1,102 (92.37)	236 (95.55)	
Yes	102 (7.08)	91 (7.63)	11 (4.45)	
Mannitol, *n* (%)				< 0.001
No	1,405 (97.57)	1,176 (98.58)	229 (92.71)	
Yes	35 (2.43)	17 (1.42)	18 (7.29)	
Diuretic, *n* (%)				< 0.001
No	1,172 (81.39)	1,000 (83.82)	172 (69.64)	
Yes	268 (18.61)	193 (16.18)	75 (30.36)	
Length of ICU stay, days, M (Q1, Q3)	2.86 (1.74, 5.95)	2.78 (1.70, 5.70)	3.78 (2.05, 7.52)	< 0.001
30-days follow time, days, Mean (±SD)	26.44 (±8.36)	30.00 (±0.00)	9.24 (±7.11)	< 0.001
Duarte-ePVS, Mean (±SD)	6.01 (±1.79)	5.90 (±1.70)	6.54 (±2.12)	< 0.001
Duarte-ePVS, *n* (%)				< 0.001
< 7.134	1,150 (79.86)	982 (82.31)	168 (68.02)	
≥7.134	290 (20.14)	211 (17.69)	79 (31.98)	
KH-ePVS, Mean (±SD)	1.22 (±11.54)	0.51 (±11.06)	4.62 (±13.14)	< 0.001
KH-ePVS, *n* (%)				< 0.001
< 11.458	1,192 (82.78)	1,017 (85.25)	175 (70.85)	
≥11.458	248 (17.22)	176 (14.75)	72 (29.15)	

SOFA, sequential organ failure assessment; GCS, Glasgow coma scale score; AMI, acute myocardial infarction; AKI, acute kidney injury; SPO_2_, oxyhemoglobin saturation; WBC, white blood cell; RDW, red blood cell distribution width; INR, international normalized ratio; BUN, blood urea nitrogen; ICU, intensive care unit; ePVS, estimated plasma volume status; Duarte-ePVS, ePVS based on hemoglobin and hematocrit (Duarte et al. formula); KH-ePVS, ePVS based on hematocrit, body weight, and gender (Kaplan–Hakim et al. formula)

### Relationship between ePVS and 30-day mortality among TBI patients

Figure 2 displays the confounder-adjusted non-linear relationship between Duarte-ePVS, KH-ePVS, and 30-day mortality risk. No significant non-linear relationship was observed between Duarte-ePVS levels and 30-day mortality risk (P_nonlinear_ = 0.730, P_overall_ = 0.915; Figure 2A). The non-linear association between KH-ePVS and 30-day mortality risk was also not significant (P_nonlinear_ = 0.063, P_overall_ = 0.005). However, when KH-ePVS levels rise to a certain level, the 30-day mortality risk presents a rapid upward trend (Figure 2B).

**Figure 2:**
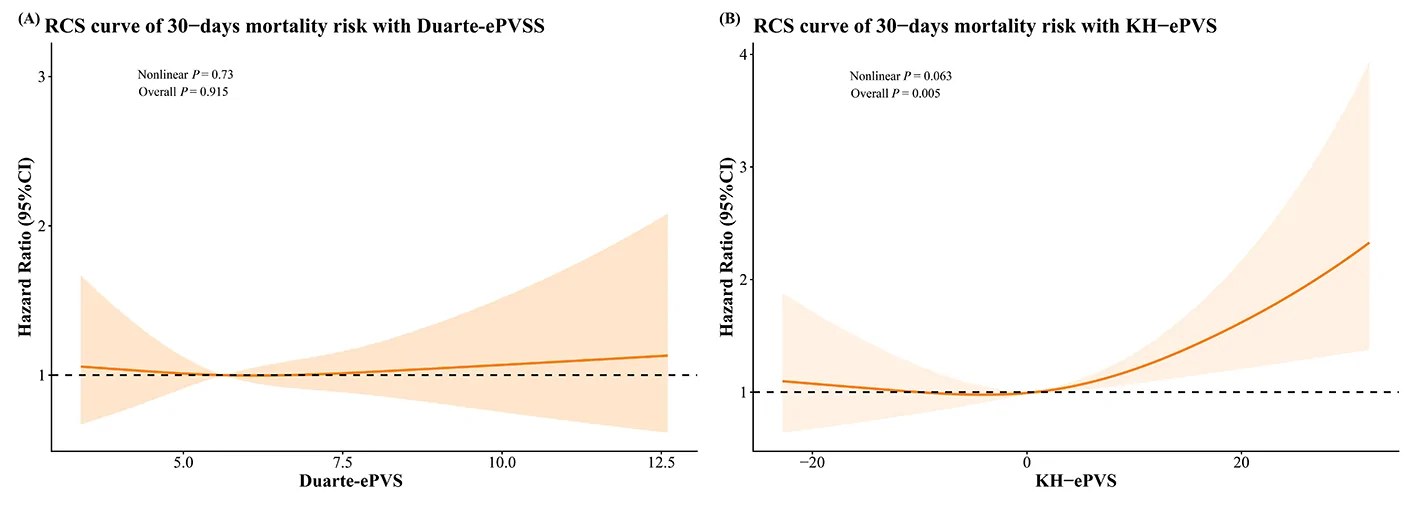
The non-linear relationship between ePVS and 30-day mortality risk among TBI patients. **(A)** Duarte-ePVS levels and 30-day mortality; **(B)** KH-ePVS levels and 30-day mortality. ePVS, estimated plasma volume status; TBI, traumatic brain injury; RCS, the restricted cubic splines.

The Cox regression analyses of the relationship between Duarte-ePVS, KH-ePVS, and 30-day mortality risk among TBI patients are shown in Table 2. A 1-SD increase in Duarte-ePVS levels [HR = 1.30 (1.17–1.44)] was associated with an elevated 30-day mortality risk in univariable analysis, and Duarte-ePVS levels ≥7.134 [HR = 1.95 (1.49–2.55)] also displayed a higher risk of 30-day mortality vs. Duarte-ePVS levels < 7.134. However, continuous Duarte-ePVS levels (*P* = 0.813) and Duarte-ePVS levels ≥7.134 (*P* = 0.449) were not associated with 30-day mortality risk in multivariable analyses. For KH-ePVS, high continuous KH-ePVS levels [unadjusted: HR = 1.37 (1.21–1.54); adjusted: HR = 1.19 (1.04–1.36)] and KH-ePVS levels ≥11.458 [unadjusted: HR = 2.10 (1.60–2.76); adjusted: HR = 1.47 (1.08–1.99)] were linked to an elevated risk of 30-day mortality both in univariable and multivariable analyses.

**Table 2:** The Cox regression analyses of the relationship between ePVS and 30-day mortality risk among TBI patients.

Variables	Univariable		Multivariable	
	HR (95% CI)	P	HR (95% CI)	P
Duarte-ePVS	1.30 (1.17–1.44)	< 0.001	1.02 (0.89–1.16)	0.813
Duarte-ePVS				
< 7.134	Ref		Ref	
≥7.134	1.95 (1.49–2.55)	< 0.001	1.13 (0.82–1.55)	0.449
KH-ePVS	1.37 (1.21–1.54)	< 0.001	1.19 (1.04–1.36)	0.012
KH-ePVS				
< 11.458	Ref		Ref	
≥11.458	2.10 (1.60–2.76)	< 0.001	1.47 (1.08–1.99)	0.015

TBI, traumatic brain injury; ePVS, estimated plasma volume status; Duarte-ePVS, ePVS based on hemoglobin and hematocrit (Duarte et al. formula); KH-ePVS, ePVS based on hematocrit, body weight, and gender (Kaplan–Hakim et al. formula); HR, hazard ratio; CI, confidence interval; Ref, reference; The multivariable Cox regression model was adjusted for age, race, SOFA, GCS, hematological system diseases, heart rate, temperature, WBC, RDW, BUN, glucose, ventilation, vasopressor, and mannitol.

A sensitivity analysis was performed by further adjusting for hematocrit, weight, and gender based on the original multivariable model (Supplementary Table 4). After additional adjustment for hematocrit, weight, and gender, continuous KH-ePVS levels [HR = 2.40 (1.59–3.61)] remained associated with the risk of 30-day mortality. However, the association between KH-ePVS levels ≥11.458 [HR = 1.46 (0.96–2.21), *P* = 0.073] and 30-day mortality risk was no longer statistically significant. Multicollinearity was assessed using variance inflation factor (VIF; Supplementary Table 5). In the Duarte-ePVS multivariable model, hematocrit (VIF = 10.97) and Duarte-ePVS (VIF = 11.63) exhibited severe multicollinearity. In the KH-ePVS multivariable model, moderate multicollinearity was observed for hematocrit (VIF = 8.81) and weight (VIF = 5.57), whereas KH-ePVS (VIF = 13.55) showed severe multicollinearity. These analyses indicated that derived variables (Duarte-ePVS or KH-ePVS) and their source variable (e.g., hematocrit, weight) should not be incorporated into the same model simultaneously.

Since many patients were excluded owing to an ICU stay of less than 24 h, a sensitivity analysis was conducted that included patients with an ICU stay exceeding 12 h (*n* = 1,779). High continuous KH-ePVS levels [HR = 1.19 (1.05–1.34)] and KH-ePVS levels ≥11.458 [HR = 1.44 (1.09–1.92)] were still associated with an increased risk of 30-day mortality (Supplementary Table 6).

Data from the MIMIC-III database (*n* = 438) were used to validate the association between ePVS and the risk of 30-day mortality (Supplementary Table 7). Duarte-ePVS levels ≥7.134 [unadjusted: HR = 1.56 (1.02–2.39)] and KH-ePVS levels ≥11.458 [unadjusted: HR = 1.73 (1.15–2.60)] were related to a higher risk of 30-day mortality in univariable analysis, but these associations were not significant in multivariable analysis (*P* > 0.05).

The influence of Duarte-ePVS and KH-ePVS on 30-day survival among TBI patients is shown in Figure 3. Both Duarte-ePVS levels ≥7.134 (vs. < 7.134) and KH-ePVS levels ≥11.458 (vs. < 11.458) presented poor survival probabilities among TBI patients (all P_log − rank_ < 0.0001). Moreover, patients in the KH-ePVS levels ≥11.458 group exhibited higher 24-h fluid balance values [1,325.25 (474.12, 2,835.11) vs. 1,043.84 (156.64, 2,229.88)] and a greater proportion of positive 24-h fluid balance (>0) [82.26 vs. 78.61%] compared with those in the KH-ePVS < 11.458 group (Supplementary Table 8).

**Figure 3:**
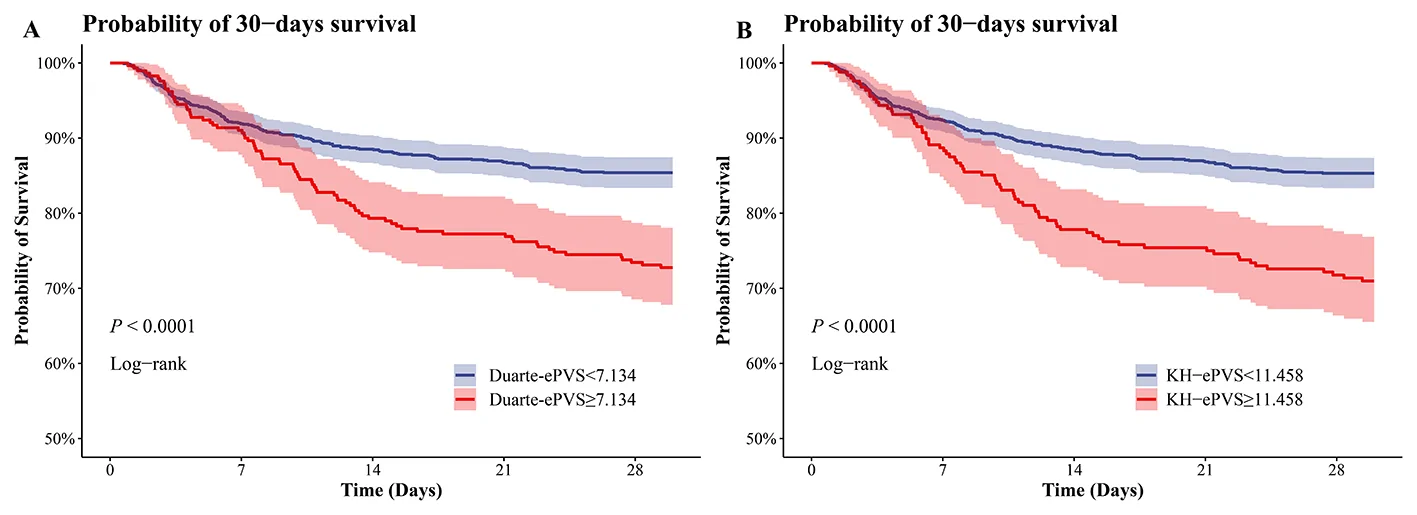
The Kaplan-Meier (KM) curves between ePVS and 30-day mortality among TBI patients. **(A)** Duarte-ePVS levels and 30-day mortality; **(B)** KH-ePVS levels and 30-day mortality. ePVS, estimated plasma volume status; TBI, traumatic brain injury.

The predictive performance of Duarte-ePVS and KH-ePVS for 30-day mortality risk was assessed. The Duarte-ePVS-combined SOFA [C-index: 0.694 (95%CI: 0.660–0.728)] and KH-ePVS-combined SOFA [C-index: 0.703 (95%CI: 0.670–0.736)] exhibited higher C-index values for predicting 30-day mortality risk than SOFA alone [C-index: 0.685 (95%CI: 0.651–0.720); Supplementary Table 9]. The Duarte-ePVS-combined SOFA [AUC: 0.713 (95%CI: 0.677–0.749)] and KH-ePVS-combined SOFA [AUC: 0.721 (95%CI: 0.686–0.756)] also showed higher AUC (the area under the receiver operating characteristic curve) values for predicting 30-day mortality risk than SOFA alone [AUC: 0.699 (95%CI: 0.662–0.736)] (Supplementary Table 10).

Figure 4 lists the association of KH-ePVS levels with 30-day mortality risk in different subgroups. These exploratory analyses suggest that KH-ePVS levels ≥11.458 exhibited a higher risk of 30-day mortality across subgroups: age [ < 65 years: HR = 1.85 (1.01–3.38); ≥65 years: HR = 1.47 (1.02–2.12)], males [HR = 1.65 (1.11–2.46)], non-heart failure [HR = 1.56 (1.10–2.20)], non-sepsis [HR = 1.67 (1.18–2.36)], non-AKI [HR = 2.36 (1.53–3.65)], and hematological system diseases [with: HR = 1.61 (1.08–2.41); without: HR = 1.89 (1.17–3.05)]. Furthermore, there may be an interaction between AKI and KH-ePVS levels on 30-day mortality risk (P_interaction_ = 0.005).

**Figure 4:**
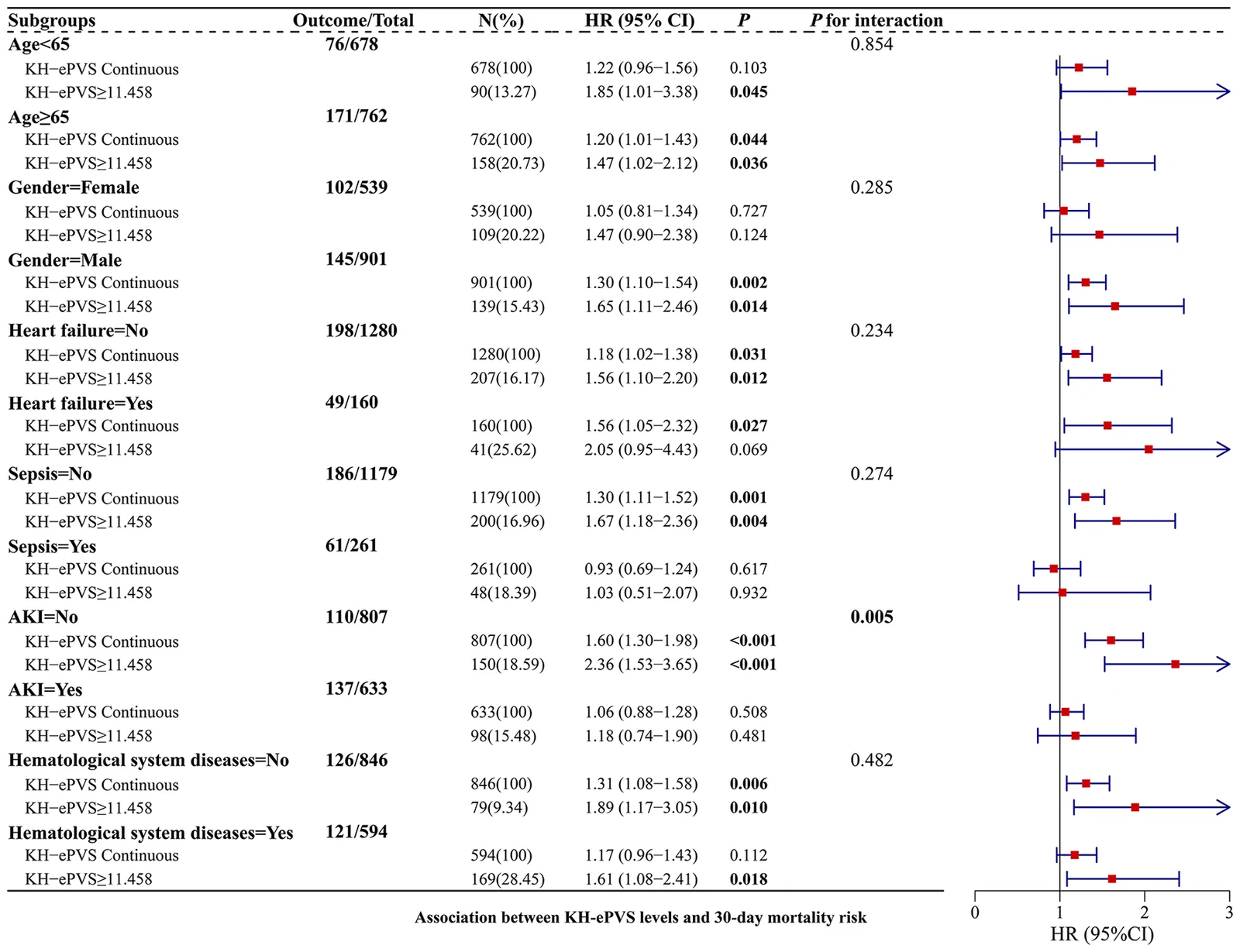
The association between KH-ePVS levels and 30-day mortality risk in different subgroups. ePVS, estimated plasma volume status; AKI, acute kidney injury.

## Discussion

The association of ePVS with 30-day mortality risk among TBI patients was examined. Elevated KH-ePVS levels were linked to a higher 30-day mortality risk among TBI patients, but Duarte-ePVS levels were not significantly related to 30-day mortality risk. Sensitivity analyses indicated that the association between KH-ePVS and the risk of 30-day mortality was robust.

TBI caused by extracranial injury may result in systemic organ dysfunction, with systemic complications covering respiratory failure, renal failure, adrenal cortical insufficiency, myocardial injury, and potential multiple organ dysfunction (2). The ICU management for TBI primarily involves monitoring intracranial pressure and cerebral perfusion pressure (21). For ICU patients, fluid management is essential to restore cardiac output, systemic blood pressure, and renal perfusion. Fluid overload can cause complications such as pulmonary edema, cerebral edema, and increased intracranial pressure (22, 23). Fluid therapy using different intravenous infusions to maintain adequate organ tissue perfusion and oxygenation is critical for critically ill TBI patients (9). A prospective study indicated that a mean positive daily fluid balance (input > loss) was linked to higher ICU mortality and poorer functional recovery (9). The current analysis explored the association of ePVS with 30-day mortality risk among TBI patients. PV is related to the regulation between the interstitial and intravascular spaces and can be used as a marker of volume overload (24). Our results revealed that high KH-ePVS levels were associated with an increased 30-day mortality risk among TBI patients, but no significant association between Duarte-ePVS levels and 30-day mortality risk was found. Serum hemoglobin, blood glucose, base excess, and mean arterial pressure have been reported to influence early mortality in patients admitted to a neuro-ICU following TBI (25). Previous studies also reported that high ePVS levels were related to an elevated all-cause mortality risk in the general population (26) and short-term mortality risk in intracerebral hemorrhage patients (17). KH-ePVS and Duarte-ePVS represent two main PVS estimation formulas. Although good correlations between different formulas have been reported, there are differences in the correlations between different formulas and diseases (27–29). He et al. indicated that KH-ePVS is superior to Duarte-ePVS in predicting contrast-induced nephropathy among heart failure patients (27). Age plays a significant role in the prognostic management of patients with TBI (30). Due to age-related declines in physiological reserve, impaired fluid regulation, pre-existing chronic conditions, and long-term medication use, elderly TBI patients are more likely to present with abnormal laboratory findings upon admission (30); these factors may affect post-traumatic circulatory volume homeostasis. KH-ePVS levels may serve as a promising indicator for monitoring admission-related physiological abnormalities in elderly patients after TBI.

The mechanism by which elevated ePVS levels increase the short-term mortality risk among TBI patients remains unclear. Elevated ePVS indicates volume overload, which is caused by plasma volume expansion and can worsen cardiac function (24). High levels of ePVS may cause excessive activation of the renin-angiotensin-aldosterone system, which may result in cardiac remodeling and a subsequent decline in cardiac function (31, 32). Overactivation of neurohormones leads to water and sodium retention, which in turn causes hemodynamic congestion and extravascular edema (33, 34). Systemic congestion may be linked to progressive edema of brain tissue, which may lead to increased interstitial pressure and reduced collateral flow in the penumbra, ultimately resulting in collateral failure and hemorrhage in patients (12, 35). Fluid overload and increased central venous pressure may generate capillary hydrostatic pressure, leading to fluid accumulation in the brain stroma, which in turn increases traumatic cerebral edema and promotes disruption of the blood-brain barrier (9, 36). Volume overload is not only related to systemic congestion but also to atrial interference with inflamatosomes, which may lead to increased cerebral infarction volume and hemorrhage conversion (37). Animal studies have shown that volume overload is linked to increased oxidative stress, stiffness, and apoptosis in cardiomyocytes (38). Fluid overload increases arterial stiffness by elevating arterial dilation (39). Furthermore, complications caused by volume overload are also a major reason for increased mortality risk among TBI patients.

This analysis examined the correlation between ePVS calculated using different methods and the short-term mortality risk of TBI patients in the ICU. The sensitivity analysis further validated the reliability of the main findings of this study. However, validation using data from the MIMIC-III database revealed that the association between ePVS and the risk of 30-day mortality was not statistically significant in multivariate analysis. One possible explanation for the non-significant finding from multivariable analysis is the relatively small sample size of the validation cohort. Besides, inclusion of a wide array of confounders in the multivariable model may weaken statistical power, hindering the detection of associations linking exposure to outcome. Nevertheless, several limitations should be acknowledged. First, the analysis data are derived from retrospective data from a single center, which inevitably introduces selection bias that affects the results. Subsequent prospective data from multiple centers are needed to further validate the research results. Second, some disease characteristics, such as the location and severity of brain damage, cannot be obtained due to database limitations, which prevents analysis based on the location of brain damage and the severity of TBI. Third, the calculation of ePVS is based on the first measurement data within 24 h after admission to the ICU, which cannot reflect the changes of ePVS over time. Fourth, significant differences in multiple characteristics existed between included and excluded patients, which may introduce selection bias in the study population.

## Conclusions

High KH-ePVS levels were linked to an increased risk of 30-day mortality among TBI patients, but not to Duarte-ePVS levels. Furthermore, it is necessary to further explore the prognostic predictive value of KH-ePVS for TBI patients, providing tools for risk stratification and prognostic management.

## Data Availability

The raw data supporting the conclusions of this article will be made available by the authors, without undue reservation.
