# Supplementary material for: Estimated plasma volume status and the risk of 30-day mortality among patients with traumatic brain injury: analysis from the MIMIC-IV database
Source: Front Neurol. 2026 Sep 14;17:1893304. doi: 10.3389/fneur.2026.1893304 (PMC13616627; doi:10.3389/fneur.2026.1893304)
Supplement: Supplementary file 1 [file Table_1.docx]

**Supplementary Table 1**. Comparative assessments of variable differences before versus after data imputation.

| Variables | Missing, n (%) | Observed data before imputation | Completed data after imputation |
| --- | --- | --- | --- |
| SPO2, M (Q₁, Q₃) | 1 (0.07%) | 99.00 (96.00, 100.00) | 99.00 (96.00, 100.00) |
| Creatinine, M (Q₁, Q₃) | 2 (0.14%) | 0.80 (0.70, 1.10) | 0.80 (0.70, 1.10) |
| Anion gap, Mean (±SD) | 2 (0.14%) | 14.54 (±3.78) | 14.54 (±3.77) |
| Temperature, Mean (±SD) | 3 (0.21%) | 36.80 (±0.84) | 36.79 (±0.84) |
| BUN, M (Q₁, Q₃) | 3 (0.21%) | 15.00 (11.00, 21.00) | 15.00 (11.00, 21.00) |
| Glucose, M (Q₁, Q₃) | 3 (0.21%) | 125.00 (104.00, 154.00) | 125.00 (104.00, 154.00) |
| Calcium, Mean (±SD) | 27 (1.88%) | 8.42 (±0.82) | 8.42 (±0.82) |
| Respiratory rate, Mean (±SD) | 56 (3.89%) | 18.61 (±5.15) | 18.63 (±5.08) |
| Fluid balance 24h, M (Q₁, Q₃) | 172 (11.94%) | 1134.20 (96.86, 2460.45) | 1126.18 (181.30, 2353.15) |
| INR, M (Q₁, Q₃) | 193 (13.4%) | 1.20 (1.10, 1.30) | 1.20 (1.10, 1.30) |

Note: SPO2, oxyhemoglobin saturation; BUN, blood urea nitrogen; INR, international normalized ratio.

**Supplementary Table 2**. Screening of confounders related to 30-day mortality among TBI patients.

| Variables | Univaraible Cox regression | | Bidirectional stepwise regression | |
| --- | --- | --- | --- | --- |
|  | HR (95% CI) | *P* | HR (95% CI) | *P* |
| Age |  |  |  |  |
| <65 years | Ref |  | Ref |  |
| ≥65 years | 2.09 (1.59-2.73) | <0.001 | 2.38 (1.75-3.23) | <0.001 |
| Gender |  |  |  |  |
| Male | Ref |  |  |  |
| Female | 1.19 (0.92-1.53) | 0.188 |  |  |
| Race |  |  |  |  |
| White | Ref |  | Ref |  |
| Black | 0.66 (0.32-1.35) | 0.257 | 0.70 (0.34-1.44) | 0.330 |
| Others | 0.74 (0.46-1.19) | 0.215 | 0.78 (0.48-1.26) | 0.305 |
| Unknown | 2.11 (1.61-2.77) | <0.001 | 2.35 (1.76-3.14) | <0.001 |
| Fluid balance 24h |  |  |  |  |
| ≤0 | Ref |  |  |  |
| >0 | 0.92 (0.68-1.24) | 0.565 |  |  |
| SOFA | 1.64 (1.50-1.78) | <0.001 | 1.25 (1.08-1.44) | 0.003 |
| GCS |  |  |  |  |
| ≥13 | Ref |  | Ref |  |
| <13 | 1.50 (1.15-1.97) | 0.003 | 1.26 (0.93-1.70) | 0.132 |
| Lesion type |  |  |  |  |
| Traumatic subdural haemorrhage | Ref |  |  |  |
| Traumatic subarachnoid haemorrhage | 0.86 (0.56-1.31) | 0.485 |  |  |
| Traumatic subarachnoid haemorrhage/Traumatic subdural haemorrhage | 1.10 (0.72-1.68) | 0.653 |  |  |
| Focal brain injury | 0.78 (0.46-1.30) | 0.334 |  |  |
| Intracranial injury of other or unspecified nature | 1.30 (0.96-1.76) | 0.092 |  |  |
| Heart failure |  |  |  |  |
| No | Ref |  |  |  |
| Yes | 2.09 (1.53-2.86) | <0.001 |  |  |
| AMI |  |  |  |  |
| No | Ref |  |  |  |
| Yes | 1.64 (0.87-3.09) | 0.124 |  |  |
| Sepsis |  |  |  |  |
| No | Ref |  |  |  |
| Yes | 1.58 (1.19-2.12) | 0.002 |  |  |
| AKI |  |  |  |  |
| No | Ref |  |  |  |
| Yes | 1.67 (1.30-2.15) | <0.001 |  |  |
| Hematological system diseases |  |  |  |  |
| No | Ref |  | Ref |  |
| Yes | 1.39 (1.08-1.78) | 0.010 | 0.69 (0.52-0.91) | 0.010 |
| Heart rate | 1.17 (1.04-1.33) | 0.010 | 1.11 (0.98-1.26) | 0.114 |
| Systolic | 0.95 (0.84-1.08) | 0.444 |  |  |
| Diastolic | 0.85 (0.74-0.97) | 0.015 |  |  |
| Respiratory rate | 1.08 (0.96-1.22) | 0.206 |  |  |
| SPO2 | 1.09 (0.95-1.25) | 0.221 |  |  |
| Temperature | 0.70 (0.64-0.76) | <0.001 | 0.76 (0.68-0.84) | <0.001 |
| WBC | 1.13 (1.07-1.19) | <0.001 | 1.08 (1.01-1.15) | 0.021 |
| Platelet | 0.95 (0.83-1.09) | 0.464 |  |  |
| RDW | 1.41 (1.29-1.54) | <0.001 | 1.31 (1.17-1.47) | <0.001 |
| Creatinine | 1.21 (1.11-1.31) | <0.001 |  |  |
| INR | 1.37 (1.25-1.50) | <0.001 |  |  |
| BUN | 1.40 (1.28-1.53) | <0.001 | 1.12 (1.00-1.26) | 0.046 |
| Glucose | 1.39 (1.27-1.52) | <0.001 | 1.18 (1.06-1.31) | 0.003 |
| Calcium | 0.89 (0.78-1.00) | 0.057 |  |  |
| Anion gap | 1.30 (1.15-1.46) | <0.001 |  |  |
| Ventilation |  |  |  |  |
| No | Ref |  | Ref |  |
| Yes | 2.79 (1.82-4.29) | <0.001 | 1.53 (0.98-2.39) | 0.064 |
| Vasopressor |  |  |  |  |
| No | Ref |  | Ref |  |
| Yes | 3.25 (2.51-4.20) | <0.001 | 1.60 (1.15-2.22) | 0.005 |
| Neurosurgical intervention |  |  |  |  |
| No | Ref |  |  |  |
| Yes | 0.57 (0.31-1.05) | 0.072 |  |  |
| Mannitol |  |  |  |  |
| No | Ref |  | Ref |  |
| Yes | 4.41 (2.73-7.14) | <0.001 | 4.26 (2.56-7.09) | <0.001 |
| Diuretic |  |  |  |  |
| No | Ref |  |  |  |
| Yes | 2.01 (1.54-2.64) | <0.001 |  |  |

Note: TBI, traumatic brain injury; SOFA, Sequential Organ Failure Assessment; GCS, Glasgow Coma Scale score; AMI, acute myocardial infarction; AKI, acute kidney injury; SPO2, oxyhemoglobin saturation; WBC, white blood cell; RDW, red blood cell distribution width; INR, international normalized ratio; BUN, blood urea nitrogen; HR, hazard ratio; CI, confidence interval; Ref, reference.

**Supplementary Table 3**. Comparison of characteristics between the included cohort and the excluded cohort.

| Variables | Total (N=2821) | Group | | *P* |
| --- | --- | --- | --- | --- |
|  |  | Excluded cohort (N=1381) | Included cohort (N=1440) |  |
| Age, n (%) |  |  |  | <0.001 |
| <65 | 1226 (43.46) | 548 (39.68) | 678 (47.08) |  |
| ≥65 | 1595 (56.54) | 833 (60.32) | 762 (52.92) |  |
| Gender, n (%) |  |  |  | 0.123 |
| Male | 1725 (61.15) | 824 (59.67) | 901 (62.57) |  |
| Female | 1096 (38.85) | 557 (40.33) | 539 (37.43) |  |
| Race, n (%) |  |  |  | 0.001 |
| White | 1779 (63.06) | 907 (65.68) | 872 (60.56) |  |
| Black | 145 (5.14) | 66 (4.78) | 79 (5.49) |  |
| Others | 370 (13.12) | 191 (13.83) | 179 (12.43) |  |
| Unknown | 527 (18.68) | 217 (15.71) | 310 (21.53) |  |
| Fluid balance 24h, ml, M (Q₁, Q₃) | 1042.70 (81.62, 2200.28) | 896.02 (36.17, 1787.10) | 1134.20 (96.86, 2460.45) | <0.001 |
| SOFA, score, Mean (±SD) | 3.07 (±2.43) | 2.56 (±2.15) | 3.56 (±2.58) | <0.001 |
| GCS, Mean (±SD) | 13.34 (±2.46) | 13.51 (±2.27) | 13.18 (±2.62) | <0.001 |
| Lesion type, n (%) |  |  |  | 0.019 |
| Traumatic subdural haemorrhage | 988 (35.02) | 524 (37.94) | 464 (32.22) |  |
| Traumatic subarachnoid haemorrhage | 410 (14.53) | 199 (14.41) | 211 (14.65) |  |
| Traumatic subarachnoid haemorrhage/Traumatic subdural haemorrhage | 320 (11.34) | 151 (10.93) | 169 (11.74) |  |
| Focal brain injury | 269 (9.54) | 130 (9.41) | 139 (9.65) |  |
| Intracranial injury of other or unspecified nature | 834 (29.56) | 377 (27.30) | 457 (31.74) |  |
| Heart failure, n (%) |  |  |  | 0.691 |
| No | 2500 (88.62) | 1220 (88.34) | 1280 (88.89) |  |
| Yes | 321 (11.38) | 161 (11.66) | 160 (11.11) |  |
| AMI, n (%) |  |  |  | 0.342 |
| No | 2755 (97.66) | 1353 (97.97) | 1402 (97.36) |  |
| Yes | 66 (2.34) | 28 (2.03) | 38 (2.64) |  |
| Sepsis, n (%) |  |  |  | <0.001 |
| No | 2463 (87.31) | 1284 (92.98) | 1179 (81.88) |  |
| Yes | 358 (12.69) | 97 (7.02) | 261 (18.12) |  |
| AKI, n (%) |  |  |  | <0.001 |
| No | 1754 (62.18) | 947 (68.57) | 807 (56.04) |  |
| Yes | 1067 (37.82) | 434 (31.43) | 633 (43.96) |  |
| Hematological system diseases, n (%) |  |  |  | <0.001 |
| No | 1775 (62.92) | 929 (67.27) | 846 (58.75) |  |
| Yes | 1046 (37.08) | 452 (32.73) | 594 (41.25) |  |
| Weight, kg, Mean (±SD) | 76.44 (±19.85) | 75.17 (±20.23) | 76.91 (±19.69) | 0.085 |
| Heart rate, bpm, Mean (±SD) | 84.52 (±18.31) | 83.60 (±17.79) | 85.41 (±18.75) | 0.008 |
| Systolic, mmHg, Mean (±SD) | 131.79 (±23.09) | 132.27 (±23.20) | 131.34 (±22.98) | 0.285 |
| Diastolic, mmHg, Mean (±SD) | 72.54 (±17.12) | 72.67 (±17.21) | 72.42 (±17.03) | 0.707 |
| Respiratory rate, bpm, Mean (±SD) | 18.46 (±4.96) | 18.31 (±4.98) | 18.61 (±4.94) | 0.116 |
| SPO2, %, Mean (±SD) | 97.47 (±2.95) | 97.27 (±2.87) | 97.65 (±3.02) | 0.001 |
| Temperature, ℃, M (Q₁, Q₃) | 36.83 (36.50, 37.17) | 36.83 (36.56, 37.11) | 36.83 (36.50, 37.17) | 0.951 |
| Height, cm, Mean (±SD) | 168.31 (±12.93) | 185.40 (±10.75) | 165.87 (±11.52) | 0.041 |
| WBC, K/uL, M (Q₁, Q₃) | 10.00 (7.50, 13.10) | 9.40 (7.10, 12.20) | 10.50 (7.80, 13.70) | <0.001 |
| Platelet, K/uL, M (Q₁, Q₃) | 191.00 (148.00, 241.00) | 193.00 (152.00, 240.00) | 189.00 (145.00, 241.00) | 0.111 |
| Hemoglobin, mg/dL, Mean (±SD) | 11.57 (±2.02) | 11.69 (±2.02) | 11.47 (±2.01) | 0.006 |
| RDW, %, Mean (±SD) | 14.13 (±1.85) | 14.12 (±1.90) | 14.15 (±1.82) | 0.673 |
| Hematocrit, %, Mean (±SD) | 34.75 (±5.75) | 35.08 (±5.75) | 34.48 (±5.74) | 0.008 |
| Creatinine, mg/dL, M (Q₁, Q₃) | 0.90 (0.70, 1.10) | 0.90 (0.70, 1.10) | 0.80 (0.70, 1.10) | 0.646 |
| INR, ratio, Mean (±SD) | 1.23 (±0.24) | 1.21 (±0.23) | 1.24 (±0.25) | 0.013 |
| BUN, mg/dL, Mean (±SD) | 18.29 (±11.84) | 18.24 (±11.81) | 18.32 (±11.88) | 0.861 |
| Glucose, mg/dL, Mean (±SD) | 134.52 (±52.21) | 132.03 (±53.14) | 136.63 (±51.33) | 0.024 |
| Calcium, mg/dL, Mean (±SD) | 8.51 (±0.75) | 8.62 (±0.72) | 8.41 (±0.77) | <0.001 |
| Anion gap, mEq/L, Mean (±SD) | 14.51 (±3.56) | 14.49 (±3.52) | 14.53 (±3.60) | 0.809 |
| Ventilation, n (%) |  |  |  | <0.001 |
| No | 965 (34.21) | 661 (47.86) | 304 (21.11) |  |
| Yes | 1856 (65.79) | 720 (52.14) | 1136 (78.89) |  |
| Vasopressor, n (%) |  |  |  | <0.001 |
| No | 2447 (86.74) | 1261 (91.31) | 1186 (82.36) |  |
| Yes | 374 (13.26) | 120 (8.69) | 254 (17.64) |  |
| Neurosurgical intervention, n (%) |  |  |  | 0.216 |
| No | 2638 (93.51) | 1300 (94.13) | 1338 (92.92) |  |
| Yes | 183 (6.49) | 81 (5.87) | 102 (7.08) |  |
| Mannitol, n (%) |  |  |  | 0.026 |
| No | 2769 (98.16) | 1364 (98.77) | 1405 (97.57) |  |
| Yes | 52 (1.84) | 17 (1.23) | 35 (2.43) |  |
| Diuretic, n (%) |  |  |  | <0.001 |
| No | 2454 (86.99) | 1282 (92.83) | 1172 (81.39) |  |
| Yes | 367 (13.01) | 99 (7.17) | 268 (18.61) |  |
| Length of ICU stay, days, M (Q₁, Q₃) | 2.01 (1.00, 4.28) | 0.99 (0.70, 2.83) | 2.86 (1.74, 5.95) | <0.001 |
| 30-days follow time, days, Mean (±SD) | 26.28 (±8.80) | 26.12 (±9.23) | 26.44 (±8.36) | 0.340 |
| 30-day follow-up, n (%) |  |  |  | 0.762 |
| Survival | 2344 (83.09) | 1151 (83.35) | 1193 (82.85) |  |
| Death | 477 (16.91) | 230 (16.65) | 247 (17.15) |  |
| Duarte-ePVS, Mean (±SD) | 5.94 (±1.77) | 5.84 (±1.74) | 6.01 (±1.79) | 0.012 |
| KH-ePVS, Mean (±SD) | 0.58 (-7.11, 8.33) | 0.33 (-7.58, 7.59) | 0.76 (-7.04, 8.39) | 0.329 |

Note: SOFA, Sequential Organ Failure Assessment; GCS, Glasgow Coma Scale score; AMI, acute myocardial infarction; AKI, acute kidney injury; SPO2, oxyhemoglobin saturation; WBC, white blood cell; RDW, red blood cell distribution width; INR, international normalized ratio; BUN, blood urea nitrogen; ICU, intensive care unit; ePVS, estimated plasma volume status; Duarte-ePVS, ePVS based on hemoglobin and hematocrit (Duarte et al. formula); KH-ePVS, ePVS based on hematocrit, body weight, and gender (Kaplan–Hakim et al. formula).

**Supplementary Table 4**. Sensitivity analysis of the association between ePVS and 30-day mortality risk among TBI patients.

| Variables | Univariable | | Multivariable | | Model 3 | |
| --- | --- | --- | --- | --- | --- | --- |
|  | HR (95% CI) | *P* | HR (95% CI) | *P* | HR (95% CI) | *P* |
| Duarte-ePVS | 1.30 (1.17-1.44) | <0.001 | 1.02 (0.89-1.16) | 0.813 | 1.24 (0.87-1.76) | 0.233 |
| Duarte-ePVS |  |  |  |  |  |  |
| <7.134 | Ref |  | Ref |  | Ref |  |
| ≥7.134 | 1.95 (1.49-2.55) | <0.001 | 1.13 (0.82-1.55) | 0.449 | 1.36 (0.88-2.10) | 0.170 |
| KH-ePVS | 1.37 (1.21-1.54) | <0.001 | 1.19 (1.04-1.36) | 0.012 | 2.40 (1.59-3.61) | <0.001 |
| KH-ePVS |  |  |  |  |  |  |
| <11.458 | Ref |  | Ref |  | Ref |  |
| ≥11.458 | 2.10 (1.60-2.76) | <0.001 | 1.47 (1.08-1.99) | 0.015 | 1.46 (0.96-2.21) | 0.073 |

Note: TBI, traumatic brain injury; ePVS, estimated plasma volume status; Duarte-ePVS, ePVS based on hemoglobin and hematocrit (Duarte et al. formula); KH-ePVS, ePVS based on hematocrit, body weight, and gender (Kaplan–Hakim et al. formula); HR, hazard ratio; CI, confidence interval; Ref, reference;

The multivariable Cox regression model was adjusted for age, race, SOFA, GCS, hematological system diseases, heart rate, temperature, WBC, RDW, BUN, glucose, ventilation, vasopressor, and mannitol.

Model 3 was further adjusted for hematocrit, weight, and gender based on the multivariable Cox regression model.

**Supplementary Table 5**. Test for multicollinearity among variables.

| Variables | Duarte-ePVS multivariable model | KH-ePVS multivariable model |
| --- | --- | --- |
|  | VIF | VIF |
| Age | 1.33 | 1.34 |
| Race | 1.28 | 1.29 |
| SOFA | 2.46 | 2.33 |
| GCS | 1.25 | 1.25 |
| Hematological system diseases | 1.36 | 1.33 |
| Heartrate | 1.23 | 1.24 |
| Temperature | 1.18 | 1.16 |
| WBC | 1.06 | 1.07 |
| RDW | 1.57 | 1.47 |
| BUN | 1.53 | 1.52 |
| Glucose | 1.29 | 1.29 |
| Ventilation | 1.1 | 1.1 |
| Vasopressor | 1.61 | 1.63 |
| Mannitol | 1.13 | 1.13 |
| Hematocrit | 10.97 | 8.81 |
| Weight | 1.35 | 5.57 |
| Gender | 1.38 | 2.15 |
| Duarte-ePVS | 11.63 | - |
| KH-ePVS | - | 13.55 |

Note: VIF, variance inflation factor; SOFA, Sequential Organ Failure Assessment; GCS, Glasgow Coma Scale score; WBC, white blood cell; RDW, red blood cell distribution width; BUN, blood urea nitrogen; ePVS, estimated plasma volume status; Duarte-ePVS, ePVS based on hemoglobin and hematocrit (Duarte et al. formula); KH-ePVS, ePVS based on hematocrit, body weight, and gender (Kaplan–Hakim et al. formula);

VIF values greater than 5 indicated the presence of multicollinearity.

**Supplementary Table 6**. Sensitivity analysis of the association between ePVS and 30-day mortality risk among TBI patients (included patients with ICU stay ≥ 12 hours).

| Variables | N (%) | Univariable | | Multivariable | |
| --- | --- | --- | --- | --- | --- |
|  |  | HR (95% CI) | *P* | HR (95% CI) | *P* |
| Duarte-ePVS | 1779 (100) | 1.33 (1.21-1.46) | <0.001 | 1.01 (0.90-1.13) | 0.871 |
| Duarte-ePVS |  |  |  |  |  |
| <7.134 | 1426 (80.16) | Ref |  | Ref |  |
| ≥7.134 | 353 (19.84) | 2.01 (1.57-2.58) | <0.001 | 1.11 (0.83-1.49) | 0.472 |
| KH-ePVS | 1779 (100) | 1.31 (1.20-1.43) | <0.001 | 1.19 (1.05-1.34) | 0.006 |
| KH-ePVS |  |  |  |  |  |
| <11.458 | 1477 (83.02) | Ref |  | Ref |  |
| ≥11.458 | 302 (16.98) | 2.12 (1.64-2.74) | <0.001 | 1.44 (1.09-1.92) | 0.011 |

Note: TBI, traumatic brain injury; ePVS, estimated plasma volume status; ICU, intensive care unit; Duarte-ePVS, ePVS based on hemoglobin and hematocrit (Duarte et al. formula); KH-ePVS, ePVS based on hematocrit, body weight, and gender (Kaplan–Hakim et al. formula); HR, hazard ratio; CI, confidence interval; Ref, reference;

The multivariable Cox regression model was adjusted for age, race, SOFA, GCS, hematological system diseases, heart rate, temperature, WBC, RDW, BUN, glucose, ventilation, vasopressor, and mannitol.

**Supplementary Table 7**. External validation of the association between ePVS and 30-day mortality risk using the MIMIC-III dataset.

| Variables | N (%) | Univariable | | | | Multivariable | | | |
| --- | --- | --- | --- | --- | --- | --- | --- | --- | --- |
|  |  | Beta | S.E | HR (95% CI) | *P* | Beta | S.E | HR (95% CI) | *P* |
| Duarte-ePVS | 438 (100) | 0.137 | 0.087 | 1.15 (0.97-1.36) | 0.113 | -0.094 | 0.112 | 0.91 (0.73-1.13) | 0.401 |
| Duarte-ePVS |  |  |  |  |  |  |  |  |  |
| <7.134 | 341 (77.85) |  |  | Ref |  |  |  | Ref |  |
| ≥7.134 | 97 (22.15) | 0.443 | 0.218 | 1.56 (1.02-2.39) | 0.042 | -0.168 | 0.254 | 0.85 (0.51-1.39) | 0.508 |
| KH-ePVS | 438 (100) | 0.082 | 0.101 | 1.08 (0.89-1.32) | 0.420 | -0.094 | 0.102 | 0.91 (0.74-1.11) | 0.358 |
| KH-ePVS |  |  |  |  |  |  |  |  |  |
| <11.458 | 329 (75.11) |  |  | Ref |  |  |  | Ref |  |
| ≥11.458 | 109 (24.89) | 0.546 | 0.209 | 1.73 (1.15-2.60) | 0.009 | 0.061 | 0.231 | 1.06 (0.68-1.67) | 0.790 |

Note: ePVS, estimated plasma volume status; MIMIC-III, the Medical Information Mart for Intensive Care-III; Duarte-ePVS, ePVS based on hemoglobin and hematocrit (Duarte et al. formula); KH-ePVS, ePVS based on hematocrit, body weight, and gender (Kaplan–Hakim et al. formula); HR, hazard ratio; CI, confidence interval; S.E, standard error; Ref, reference;

The multivariable Cox regression model was adjusted for age, race, SOFA, GCS, hematological system diseases, heart rate, temperature, WBC, RDW, BUN, glucose, ventilation, vasopressor, and mannitol.

**Supplementary Table 8**. Comparison of 24-hour cumulative fluid balance among different KH-ePVS groups.

| Variables | Total (N=1440) | KH-ePVS<11.458 (N=1192) | KH-ePVS≥11.458 (N=248) | Statistics | *P* |
| --- | --- | --- | --- | --- | --- |
| Fluid balance 24h, mL, M (Q₁, Q₃) | 1101.88 (205.73, 2332.99) | 1043.84 (156.64, 2229.88) | 1325.25 (474.12, 2835.11) | W = 130891.000 | 0.005 |
| Fluid balance 24h, mL, n (%) |  |  |  | χ² = 1.448 | 0.229 |
| ≤0 | 299 (20.76) | 255 (21.39) | 44 (17.74) |  |  |
| >0 | 1141 (79.24) | 937 (78.61) | 204 (82.26) |  |  |

Note: ePVS, estimated plasma volume status; KH-ePVS, ePVS based on hematocrit, body weight, and gender (Kaplan–Hakim et al. formula).

**Supplementary Table 9**. Comparison of the C-index of SOFA and ePVS in predicting 30-day mortality in patients with TBI.

| Variables | C-index (95% CI) | Statistic | P |
| --- | --- | --- | --- |
| SOFA alone | 0.685 (0.651-0.720) | Ref |  |
| SOFA + Duarte-ePVS | 0.694 (0.660-0.728) | 2.968 | 0.003 |
| SOFA + KH-ePVS | 0.703 (0.670-0.736) | 2.459 | 0.014 |

Note: SOFA, Sequential Organ Failure Assessment; ePVS, estimated plasma volume status; TBI, traumatic brain injury; Ref, reference; CI, confidence interval; Duarte-ePVS, ePVS based on hemoglobin and hematocrit (Duarte et al. formula); KH-ePVS, ePVS based on hematocrit, body weight, and gender (Kaplan–Hakim et al. formula).

**Supplementary Table 10**. Comparison of the predictive performance of SOFA and ePVS for 30-day mortality in patients with TBI.

| Name | Accuracy (95% CI) | Specificity (95% CI) | Sensitivity (95% CI) | PPV (95% CI) | NPV (95% CI) | AUC (95% CI) | Statistic | P |
| --- | --- | --- | --- | --- | --- | --- | --- | --- |
| SOFA alone | 0.733 (0.709-0.755) | 0.769 (0.746-0.793) | 0.555 (0.493-0.617) | 0.333 (0.287-0.378) | 0.893 (0.874-0.912) | 0.699 (0.662-0.736) | Ref |  |
| SOFA + Duarte-ePVS | 0.681 (0.656-0.705) | 0.681 (0.655-0.708) | 0.676 (0.618-0.734) | 0.305 (0.267-0.344) | 0.910 (0.892-0.929) | 0.713 (0.677-0.749) | 2.96 | 0.003 |
| SOFA + KH-ePVS | 0.672 (0.647-0.696) | 0.670 (0.643-0.696) | 0.684 (0.626-0.742) | 0.300 (0.262-0.338) | 0.911 (0.892-0.930) | 0.721 (0.686-0.756) | 2.7 | 0.007 |

Note: PPV, positive predictive value; NPV, negative predictive value; AUC, the area under the receiver operating characteristic curve; SOFA, Sequential Organ Failure Assessment; ePVS, estimated plasma volume status; TBI, traumatic brain injury; Ref, reference; CI, confidence interval; Duarte-ePVS, ePVS based on hemoglobin and hematocrit (Duarte et al. formula); KH-ePVS, ePVS based on hematocrit, body weight, and gender (Kaplan–Hakim et al. formula).

**Supplementary Figure 1**. Box plot of the data distribution before and after missing data imputation. SPO2, oxyhemoglobin saturation; BUN, blood urea nitrogen; INR, international normalized ratio.
